# The Effect of Mindfulness Yoga in Children With School Refusal: A Study Protocol for an Exploratory, Cluster-Randomized, Open, Standard Care-Controlled, Multicenter Clinical Trial

**DOI:** 10.3389/fpubh.2022.881303

**Published:** 2022-07-13

**Authors:** Marie Amitani, Haruka Amitani, Tetsuhiro Owaki, Takako Monuki, Satomi Adachi, Suguru Kawazu, Takamasa Fukumoto, Hajime Suzuki, Takuya Yoshimura, Kimiko Mizuma, Yuko Nishida, Hiroko Watanabe, Masayuki Hirose, Kouta Funakoshi, Keiko Ota, Kenta Murotani, Akihiro Asakawa

**Affiliations:** ^1^Education Center for Doctors in Remote Islands and Rural Areas, Kagoshima University Graduate School of Medical and Dental Sciences, Kagoshima, Japan; ^2^Division of Community-Based Medicine, Kagoshima University Graduate School of Medical and Dental Sciences, Kagoshima, Japan; ^3^Division of Psychosomatic Internal Medicine, Kagoshima University Graduate School of Medical and Dental Sciences, Kagoshima, Japan; ^4^Multidisciplinary Pain Center, Kyushu University Hospital, Fukuoka, Japan; ^5^Division of Oral and Maxillofacial Surgery, Kagoshima University Graduate School of Medical and Dental Sciences, Kagoshima, Japan; ^6^Center for Clinical and Translational Research, Kyushu University Hospital, Fukuoka, Japan; ^7^Center for Clinical Research and Innovation, Osaka City University Hospital, Osaka, Japan; ^8^Biostatistics Center, Kurume University, Fukuoka, Japan

**Keywords:** children, school refusal, anxiety, mindfulness yoga, a cluster-randomized controlled trial

## Abstract

**Background:**

School refusal occurs in about 1–2% of young people. Anxiety and depression are considered to be the most common emotional difficulties for children who do not attend school. However, at present, no definitive treatment has been established for school refusal, although interventions such as cognitive behavioral therapy have been used. This paper reports a protocol for a cluster-randomized controlled trial of a mindfulness yoga intervention for children with school refusal.

**Methods:**

This study is a multicenter, exploratory, open cluster-randomized controlled trial. This study will recruit children aged 10–15 years with school refusal. After a 2-week baseline, participants for each cluster will be randomly assigned to one of two groups: with or without mindfulness yoga for 4 weeks. Mindfulness yoga will be created for schoolchildren for this protocol and distributed to the participants on DVD. The primary outcome is anxiety among children with school refusal using the Spence Children's Anxiety Scale-Children.

**Discussion:**

For this study, we developed a mindfulness yoga program and protocol, and examine whether mindfulness yoga can improve anxiety in children with school refusal. Our mindfulness yoga program was developed based on the opinions of children of the same age, and is a program that children can continue to do every day without getting bored. In this way, we believe that we can contribute to the smooth implementation of support to reduce the anxiety of children with school refusal, and to the reduction of the number of children who refuse to go to school.

## Highlights

- We have developed a mindfulness yoga program that children can do at home alone.
- This is the first randomized control study of a yoga intervention for children with school refusal.
- We will conduct a cluster-randomized trial to avoid children influencing each other in the facility.

## Introduction

School attendance is a basic ability that is important for children and adolescents. School refusal is a psychosocial problem characterized by difficulty attending school, and in many cases, substantial absence from school (1). In addition, school refusal has been shown to affect learning and achievement negatively and to place youth at risk for early school dropout (2, 3). School refusal occurs in about 1–2% of young people (4). Children with school attendance problems are also likely to have emotional difficulties (5). Anxiety and depression are considered the most common emotional difficulties for children who do not attend school (6, 7). In addition, Hochadel et al. (8) reported that there was a definite relationship between sleep problems and school refusal.

In Japan, school refusal is defined by the Ministry of Education, Culture, Sports, Science and Technology (MEXT) as “students who are absent from school more than 30 days a year due to some psychological, emotional, physical, or social factors or background that prevents them from attending or wanting to attend school, excluding those who are absent due to illness or financial reasons.” The number of Japanese children with school refusal has increased every year since 2012, with the highest number recorded in 2020. A survey by MEXT reported that 0.83% of elementary school students and 3.94% of junior high school students were absent from school for a long period of time (9). Possible reasons for school refusal among elementary school students include anxiety and other emotional turmoil (36.1%), apathy (23.0%), and problems with the parent–child relationship (19.1%), whereas those among junior high school students include emotional turmoil such as anxiety (28.1%), apathy (26.7%), and problems related to friendships, excluding bullying (15.4%). In addition, the proportion who report “disruption of the rhythm of life” is on the rise. Thus, anxiety and apathy (except for bullying and friendships) are the main reasons for more than 50% of school refusal cases.

While various interventions have been used to treat school refusal (e.g., psychodynamic treatment, family therapy, medication), cognitive behavioral therapy (CBT) is the most commonly studied. CBT typically involves treatment components such as psychoeducation, relaxation training, cognitive restructuring, graded exposure, and social skills training (10). However, few randomized controlled trials (RCTs) assessing the effectiveness of CBT have been reported, and its efficacy is only partial (4).

Mindfulness is defined as paying total attention to the present moment with a non-judgmental awareness of the inner and/or outer experiences (11). Originally derived from Eastern traditions and Buddhist psychology, mindfulness can be cultivated by various techniques (12, 13). Formally, it is trained by meditation practices such as sitting meditation or physical movements such as yoga. These techniques help steady the mind and train its attentional capacity while increasing its breadth of focus. In several meta-analyses, mindfulness-based interventions have proven to be effective in treating clinical and stress-related problems and disorders in various disease groups (14–16).

Yoga is one of the ṣ*aḍdarśanas* of Indian philosophy and is said to have a history of 5,000 years (17). The word “yoga” comes from the Sanskrit words for “integration” and “connection,” and aims to harmonize the mind and body, allowing a connection to be felt between oneself and one's surroundings. The Sanskrit word for “health,” “*swasthya*,” is derived from two Sanskrit words, “*swa*,” meaning “self,” and “*stha*,” meaning “stay.”(18). In other words, to be healthy is to stay in oneself, and the connection between being who you are and being healthy is expressed in ancient languages.

It is reported that 300 million people practice yoga across the globe (19). The 2012 and 2017 National Health Interview Survey reported that yoga was the most commonly practiced complementary health approach among US adults, and that the use of yoga and meditation during the past 12 months increased from 2012 to 2017 among US children aged 4-17 years (20, 21). Yoga is considered to be easy to practice for children and adults of all ages. Yoga has also been shown to be effective in improving self-esteem, emotional well-being, positive emotions, and self-regulation in adolescents (22). In several meta-analyses, yoga interventions have been proven to be effective for treating fatigue, pain, hypertension, and type II diabetes (23–26). The intentional combination of mindfulness and yoga can be powerful, resulting in increased stress tolerance and sleep quality and reduced psychological distress and chronic pain intensity (27–29).

McCall et al. reported in a systematic review that mindfulness yoga appears most effective for reducing symptoms in anxiety, depression, and pain (30). Mindfulness yoga interventions have been shown to improve sleep quality significantly among female teachers with chronic insomnia working in primary schools (31, 32). In addition, older adults with insomnia who engaged in mindfulness yoga classes twice a week for 12 weeks had better sleep quality, efficiency, latency, and duration than the control group (33).

For children, mindfulness yoga interventions have been reported to reduce stress levels (34), improve coping with anxiety and anger (35), and increase attention span (36). In a systematic review, nearly all studies indicated reduced anxiety among children and adolescents (age 3–18 years) after a mindfulness yoga intervention (37). Another systematic review recently reported that mindfulness yoga generally leads to some reductions in anxiety and depression in children and adolescents (38).

However, to our knowledge, there is a paucity of research on safe and effective therapeutic interventions for anxiety associated with school refusal. The main purpose of this study is to examine whether a mindfulness yoga intervention can improve anxiety in children with school refusal. In addition, we will examine whether this intervention can improve depressed mood, sleep rhythm, and activity levels. By conducting mindfulness yoga intervention, we will verify the usefulness and effectiveness of a safe and reliable alternative treatment for alleviating anxiety and depression in children with school refusal.

## Methods

### Study Design

This study is a multicenter, exploratory, open cluster-randomized controlled trial. A multicenter study was set up because the background of school refusal in children may be influenced by community characteristics. The study will consist of two groups: a mindfulness yoga group and a non-mindfulness yoga group, and the participants will clearly be aware of the treatment. Because of the potential for children within a facility to influence each other, we decided to conduct a cluster-randomized trial with the same intervention within the facility. Ideally, in an open trial, the primary outcome should be an objective measure. However, since the most standard measure of anxiety among children is a self-administered questionnaire, it must be subjective. Therefore, objective evaluation items were set as secondary evaluation items. We believe that this will ensure the evaluation of effectiveness.

### Mindfulness Yoga Program Development

This mindfulness yoga program aims to provide a space and time for children to be able to exist “as they are” to create the psychological place they seek. This mindfulness yoga program is a 4-week program that is done alone at home by watching a mindfulness yoga program video, and the space required for the mindfulness yoga is the size of about a normal exercise mat. Initially, 9 children were given the opportunity to experience the mindfulness yoga program. Feedback was received from the children about the mindfulness yoga program in terms of their time and ability to continue. To make it easier for the participants to continue, one program was set at 20 min or less and the program content changed every week. In the first session, the participants will watch a video in front of the researcher and do the first program. The researcher will instruct them on points such as how to breathe and how to relax. After distribution, the students will be asked to follow the instructions in the video to practice mindfulness yoga (Supplementary Figures 1A–E). Students will also be asked to keep a daily mindfulness yoga diary (Supplementary Figures 2A–D) to track their practice.

The program DVD is structured with the following points in mind:

1. The program encourages participants to explore their senses and feelings to focus on the state of mindfulness with a non-judgmental awareness of the inner and/or outer experiences, rather than to “do it well” or otherwise perfect the performance.
2. The program starts at a low intensity so that it can be implemented by children with school refusal with low activity intensity. The three major asanas and balance poses of yoga were incorporated by moving the limbs extensively, and the whole body is moved to compensate for the lack of activity.
3. Simple movements that can be safely performed by one person and humming breathing to encourage vocalization were incorporated.

### Interventions

**Figure 1:**
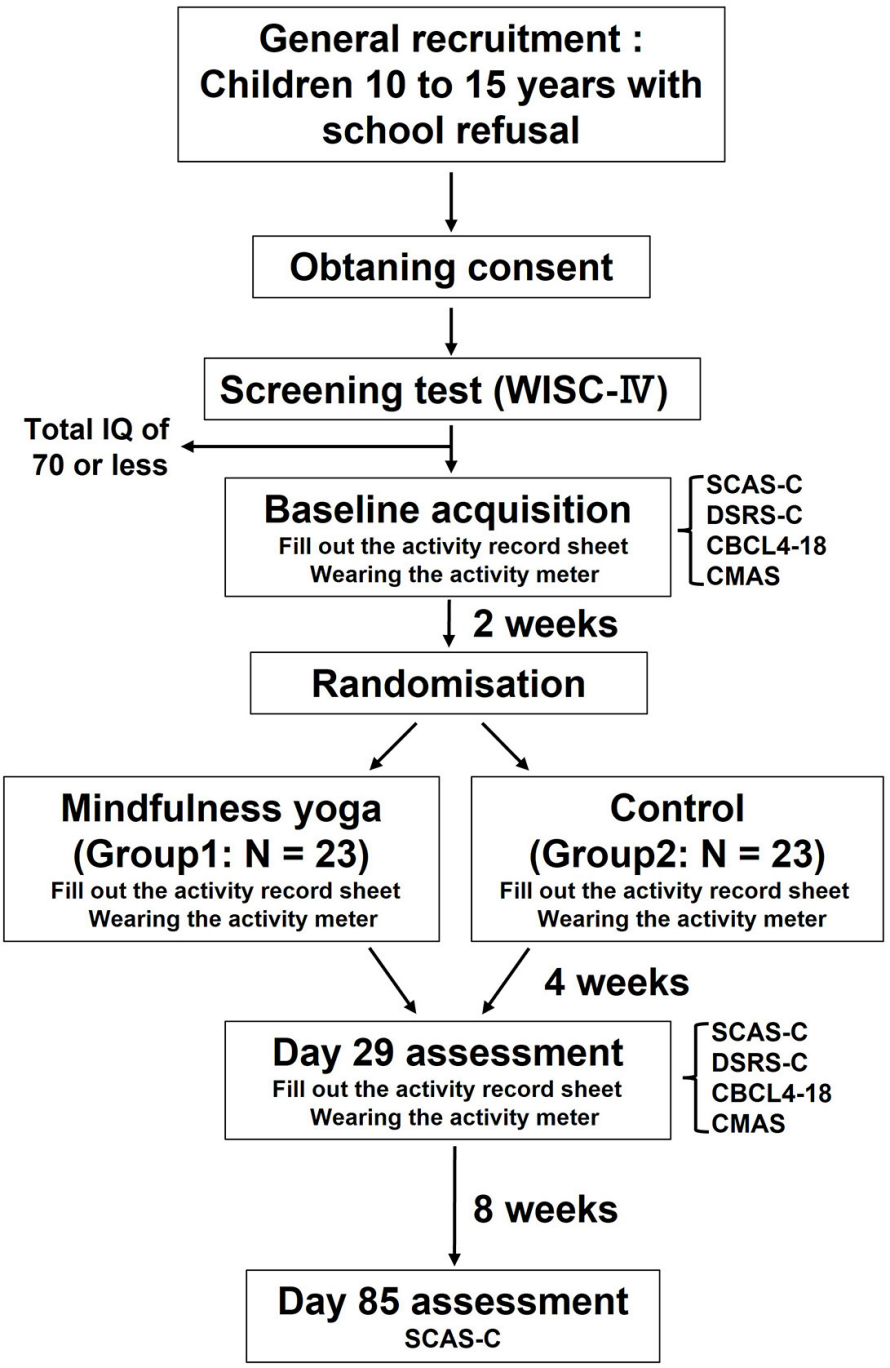
Flowchart of the study schedule. WISC-IV, Wechsler Intelligence Scale for Children, Fourth Edition; SCAS-C, Spence Children's Anxiety Scale–Children; DSRS-C, Depression Self Rating Scale for Children; CBCL-4–18, Child Behavior Checklist for ages 4–18; CMAS, Children's Manifest Anxiety Scale.

The study schedule is shown in Figure 1. Group A (Standard care + mindfulness yoga intervention group): After obtaining a 2-week baseline of activity and other data, the participants will perform mindfulness yoga for 28 days. The mindfulness yoga session will be conducted according to a video recording of the instructions. At the time of distribution of the mindfulness yoga video, the participants will perform mindfulness yoga along with the mindfulness yoga video to ensure that the researcher understands the purpose of the activity. After the end of the intervention period, the percentage of days in school will be tracked for 8 weeks. Group B (standard treatment group): After obtaining a 2-week baseline of activity and other data, the participants will continue to measure their activity and other data for 4 weeks. The progress of the percentage of days in school will be followed for another 8 weeks.

Both groups will fill out an activity record sheet for the first 14 weeks of the study and wear an actigraph. Both groups will receive 30 min of supportive psychotherapy at the start date and at 2, 6, and 14 weeks, as well as lifestyle coaching on rhythm of life using the activity record sheet. The results of intelligence and psychological tests will be used to provide feedback on innovations to be aware of in daily life and social relationships. Standard care includes self-monitoring and 30-min counseling sessions.

Patients who have been visiting a medical institution or psychological consultation office for treatment prior to participating in the study, or who are attending an alternative school, will continue to receive treatment and consultation, and will not, in principle, change their medication or treatment during the study period. If the treatment is changed during the study period, the details of the change shall be communicated to the person in charge for confirmation in the medication notebook or information form. The participants in the control group will be given the chance to receive the mindfulness yoga program after the completion of this study.

### Participants

The study participants will be children in Japan with school refusal aged 10–15 years old who are absent from school for 30 days or more per year. This study was approved by the ethical review board of Kagoshima University (IRB No: 190263). It is estimated that the study dates would be from 23 April 2020 to 31 March 2025. Study recruitment will be done through posters and other means. We will provide explanations using the consent explanatory document and consent forms, and written consent will be obtained from all participants and their guardians.

#### Inclusion Criteria

1. Children who have been absent from school for 30 days or more per year and who meet MEXT's definition of school refusal
2. Children aged 10–16 years at the time of obtaining consent
3. Children with no continuous mindfulness yoga experience or experience deemed equivalent to mindfulness yoga by the physician in charge for a period of 1 year prior to the date of the examination

#### Exclusion Criteria

1. Children with a total intelligence quotient (IQ) of 70 or less according to the Wechsler Intelligence Scale for Children, Fourth Edition (WISC-IV)
2. Children who will be age 16 years or older during the research period
3. Children judged by the physician in charge to be inappropriate for inclusion in this study because of the heavy physical and mental burden of being a participant in this research
4. Children with a history of wrist-cutting or attempted suicide
5. Children judged to have the potential for self-injury or other harm
6. Children judged to require an immediate social response, such as those exposed to abuse or bullying
7. Children who have a change in their commuting environment such as graduation, advancement to higher education, or withdrawal from school within the research period
8. Children with a history of schizophrenia, paranoid disorder, depression with thoughts of death, etc., for which the physician in charge has determined that specialized treatment is necessary
9. Children deemed inappropriate by the principal investigator, etc.

### Screening Test

The WISC-IV, which is the most widely used intelligence battery in clinical practice, will be conducted as a screening test to assess the general cognitive abilities (IQ) of the children (39). Scaled scores will be derived from index scores in accordance with normative data based on the child's age and gender [mean = 100, standard deviation (SD) = 15].

### Randomization

Cluster randomization will be conducted with facilities as clusters. A block randomization method with two strata will be used: “free schools” and “facilities other than free schools.” The allocation ratio will be 1:1.

### Primary Outcome Measures

We set the Spence Children's Anxiety Scale-Children (SCAS-C) as of the 29th day after the start of the intervention as the primary outcome measure, based on previous studies (40–42). The SCAS-C is a test based on the Diagnostic and Statistical Manual of Mental Disorders, fourth edition, text revision, that assesses anxiety in children using six subscales: separation anxiety disorder, social anxiety disorder, obsessive–compulsive disorder, panic disorder, generalized anxiety disorder, and traumatic fear (localized phobia). The SCAS-C was designed to assess anxiety in school-age children, as it has been suggested that anxiety is associated with school non-attendance in Japan (43).

### Secondary Outcome Measures

1. Percentage of school daysAs one of the objective indicators of behavioral change in school refusal, we set the percentage of school days from the day after the start of intervention until the 29th day and from the 30th to 85th days.
2. Average daily activity
3. Behavioral cycle
4. Sleep cycle
5. Average sleep efficiencyThe amount of activity was set for the purpose of improving the accuracy of the measurement and objectively capturing changes in the amount of activity as it is thought that the rhythm of life is disrupted because of school refusal, which may affect the amount of activity. In addition, as it is thought that the disruption of the daily rhythm caused by school refusal also affects sleep, the sleep cycle and sleep efficiency were established for the purpose of objective measurement. All participants will be asked to fill out an activity record sheet (Supplementary Figure 3) to determine their activity level, life/sleep rhythm and school attendance. In addition, they will be asked to wear a sleep analyzer (Sleep-Sign-Act, version 2.0; Kissei Comtec, Nagano, Japan), which has been used in a number of previous studies, including those with children (44–46), to measure objective activity levels and sleep quality.
6. Body temperature
7. Systolic blood pressure
8. Diastolic blood pressure
9. Number of pulsed beats
10. Height and weight
11. Body mass indexBody temperature, blood pressure (systolic and diastolic), pulse, height and weight (only at the beginning), and body mass index were set to measure the physical effects of mindfulness yoga.
12. Depression Self Rating Scale for Children (DSRS-C)The DSRS-C is composed of 18 question items in plain language and is widely used to measure children's depressive symptoms (47). The total possible scores ranges from 0 to 36. A higher total score (>16) reflects a greater risk of clinical depression. Murata et al. modified a Japanese version of the DSRS-C, and have demonstrated its satisfactory reliability and validity. In addition, the DSRS-C was established because it has been suggested to be a powerful tool for understanding children's mentalities and has high discriminative accuracy as a screening test (48).
13. Child Behavior Checklist for ages 4–18 (CBCL-4-18)The CBCL-4-18 was developed by Achenbach (49) as an objective assessment tool to assess the behavioral and emotional characteristics of children. It is completed by parents and consists of approximately 100 competence items (e.g., daily activities, relationships with friends and parents, academic performance) and problematic behavior. Itani et al. modified a Japanese version of the CBCL-4-18 (50).
14. Children's Manifest Anxiety Scale (CMAS)The CMAS is a validated scale used to assess anxiety disorders in children. It is composed of 53 items, and possible scores range from 0 to 42 (51). Sakamoto et al. modified a Japanese version of the CMAS and have demonstrated its satisfactory reliability and validity (52).

### Sample Size Calculation

The intracluster correlation coefficient (ICC) ρ examined in a previous study, a randomized controlled trial of CBT for anxiety disorders in children, ranged from 0.12 to 0.22, so we assumed that the ICC in this study would be 0.17, the median of previous studies.

A pilot study was conducted in Japan to evaluate the efficacy of CBT in children with anxiety disorders using the Japanese version of the SCAS-C, and the results were as follows.


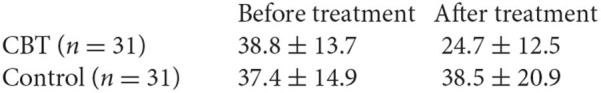


Considering that mindfulness yoga therapy in this study will have the same effect as CBT, we estimated the mean post-treatment SCAS-C score to be 24.7 in the mindfulness yoga therapy group and 38.5 in the control group (53), and calculated the population variance from the weighted mean, considering that the variances of the CBT and control groups would be equal (54).


$$\begin{array}{l} s^{2}=\frac{\left(31-1\right){\cdot 12.5}^{2}+\left(31-1\right)\cdot 20.9^{2}}{31+31-2}=17.2^{2} \end{array}$$

The SD for this study was conservatively estimated at 17.2. Assuming a one-sided significance level of 10% and a power of 80%, the number of subjects was calculated to be 30. Since this is a cluster-randomized trial, the required number of subjects (Ncluster) for a cluster-randomized trial is *Ncluser* = *N*·(1+(*r*−1)·ρ), where N is the sample size designed for a normal RCT. If the average number of subjects per center were three and the ICC ρ 0.17, *Ncluser* = 30·(1+(3−1)·0.17) = 40.2. The required number of subjects was therefore 46, considering a dropout rate of 10% (55).

### Statistical Analysis

#### Analysis Set

Statistical analysis will be performed for the following analysis sets.

#### Full Analysis Set

All participants, excluding those who were treated in serious violation of ethical guidelines and those whose data for the primary outcome were absent, will be considered the FAS.

#### Safety Analysis Set

The population for which standard treatment is administered will be defined as the SAS.

#### Per Protocol Set

The population consisting of participants who are included in the FAS and do not meet the following conditions will be defined as the PPS.

- Participants who do not meet the inclusion criteria specified for this study or who violate the exclusion criteria
- Significant deviations from the research protocol
- Participants who had not practiced yoga for more than 20 days

#### Analysis Items and Contents

Summary statistics refer to the number of cases, mean, SD, median, minimum, and maximum values.

#### Breakdown of Participants

The following tabulations will be made for the enrolled examples:

1. The number of included and excluded participants in the FAS, PPS, and SAS will be tabulated by treatment group and shown in a flowchart. For the excluded participants, a breakdown of the reasons for exclusion will be tabulated.
2. Completed and discontinued participants will be tabulated. For the discontinued participants, a breakdown of the reasons for discontinuation will be tabulated.

#### Summary Between Treatment Groups

Participant backgrounds will be tabulated by treatment group for each FAS. Summary statistics will be presented for continuous data, and frequencies and proportions (%) for categorical data.

#### Status of Mindfulness Yoga Practice

Summary statistics will be calculated for the SAS regarding the number of days and percentage of mindfulness yoga performed.

#### Analysis of the Primary Outcome

The following analysis will be conducted for the FAS. In addition, the same analysis will be conducted for the PPS as a sensitivity analysis to confirm the robustness of the results:

1. A linear mixed effect model will be conducted for the SCAS-C with baseline values and stratification factor (free school/non-free school facility) as covariates, with each facility as a random effect. Statistical testing will use a one-sided significance level of 10%. If the one-sided *p*-value is <0.1, the null hypothesis will be rejected, and we will proceed to a pivotal study, judging that efficacy of mindfulness yoga treatment has been confirmed.
2. The ICC will be calculated.
3. Summary statistics for the SCAS-C and its change by group and time point (baseline, Day 29, and Day 85) will be shown.
4. The mean and SD of the SCAS-C values will be plotted.

### Data Management

The study data will be collected and stored within the REDCap system, which is a web-based, secure, and HIPAA-compliant research management platform. REDCap fully supports several research processes for subject scheduling, subject randomization, data entry and management, as well as data safety monitoring and adverse event reporting. A particular advantage of REDCap is that it leaves a complete audit trail. In addition, the input person is immediately notified if fields are missing and/or out of range values are entered. However, in the case of a loss of Internet access, backup paper copies will be provided, which would then be subsequently entered into REDCap.

Enrolled participants will be identified and queried using REDCap, and no name or other information that would allow a third party to identify a participant directly will be entered into the study database. The data sets stored on a PC will be encrypted and a password will be set to prevent third parties from using the files. Participant identification will be done by numbers or symbols, and the correspondence between these and information that can easily identify the individual (e.g., name, address) to a third party will not be stored in the data set. A list of the correspondence between the information that can be easily identified by a third party (e.g., name, address) shall be stored in a lockable cabinet, etc., (personal information manager: MA).

### Monitoring

The principal investigator will have the institution conduct monitoring to confirm that this research is being conducted properly. In principle, the monitoring report will be submitted once a year.

### Adverse Events

There are no known health or safety risks associated with participation in the described study, and the risk of adverse events is low. Data monitoring will be monitored by the safety monitoring committee. Any minor or major events associated with the intervention or usual care groups will be monitored throughout the duration of the 1-week program. The chief investigators, MA and HA, will review any adverse events or unintended effects detected.

### Patient and Public Involvement

1. In order to develop this yoga program, we asked children between the ages of 10 and 15 for their opinions on whether the program content and time would allow them to continue every day.
2. In preparing the informed assent document, we asked children between the ages of 10 and 15 for their opinions on whether they could read the text and whether the content was easy to understand.
3. Citizen members participated in the ethical review board of Kagoshima University and exchanged opinions.

## Discussion

For this study, we developed a mindfulness yoga program and protocol, and examine whether mindfulness yoga can improve anxiety in children with school refusal. Our mindfulness yoga program was developed based on the opinions of children of the same age, and is a program that children can continue to do every day without getting bored.

This study has several strengths. First, it is a new yoga program that combines mindfulness and yoga. Second, this is incorporation of cluster randomization, in which schools and affiliations are randomized as clusters. Third, the content of mindfulness yoga program and time designed as sustained without burden by incorporating children's opinions in the study design. Fourth, because it is a video-based intervention, it can be used in a variety of settings, the ability to implement mindfulness yoga in schools and homes without being limited by geographical factors, since the video-based intervention allows mindfulness yoga to be implemented without direct instructor contact.

The study also has several limitations. First, the analysis design does not include social backgrounds that may contribute to truancy, such as parent-child relationships, economic problems, and friendships. Second, the study does not include developmental disabilities other than intellectual disabilities as the exclusion criteria. Third, the seasonal variation of school refusal was not considered in the intervention design or data collection.

To our knowledge, little research on mindfulness-yoga intervention for children with school refusal has been conducted. Our proposed study is the first RCT intervention for the children with school refusal to be implemented. If this study shows positive results, it will be possible to recommend that mindfulness yoga be implemented with similar strategies with other children and beyond. In addition, the use of mindfulness yoga programs that can be implemented at home or at school may promote cooperation between the fields of medicine and education, and provide comprehensive and integrated support for school children. In this way, we believe that we can contribute to the smooth implementation of support to reduce the anxiety of children with school refusal, and to the reduction of the number of children who refuse to go to school.

## Ethics Statement

The studies involving human participants were reviewed and approved by Kagoshima University (IRB No: 190263).

## Author Contributions

MA, TM, SA, TO, KM, and AA were responsible for the initial protocol drafting and design. HA, SK, KM, HS, TY, and YN contributed to preliminary searches used to develop the rationale and background of the study. TF and KO built the REDCap system for this study. HW, MH, and KF advised on methods design and statistical analysis techniques that could be used. All authors contributed and approved this final manuscript.

## Funding

This research was supported by AMED (Grant Nos. JP19lk0310062 and JP21lk0310067).

## Conflict of Interest

The authors declare that the research was conducted in the absence of any commercial or financial relationships that could be construed as a potential conflict of interest.

## Publisher's Note

All claims expressed in this article are solely those of the authors and do not necessarily represent those of their affiliated organizations, or those of the publisher, the editors and the reviewers. Any product that may be evaluated in this article, or claim that may be made by its manufacturer, is not guaranteed or endorsed by the publisher.

## Supplementary Material

Supplementary Figure 1A Contents of the first day of the first week of the program.

Supplementary Figure 1B Contents of days 2 through 7 of the first week of the program.

Supplementary Figure 1C Contents of days 8 through 14 of the second week of the program.

Supplementary Figure 1D Contents of days 15 through 21 of the third week of the program.

Supplementary Figure 1E Contents of days 15 through 21 of the fourth week of the program.

Supplementary Figure 2A Contents of the diary of the first week.

Supplementary Figure 2B Contents of the diary of the second week.

Supplementary Figure 2C Contents of the diary of the third week.

Supplementary Figure 2D Contents of the diary of the fourth week.

Supplementary Figure 3 An activity record sheet.
