# Supplementary figures and images for: The Effect of Mindfulness Yoga in Children With School Refusal: A Study Protocol for an Exploratory, Cluster-Randomized, Open, Standard Care-Controlled, Multicenter Clinical Trial
Source: Front Public Health. 2022 Jul 13;10:881303. doi: 10.3389/fpubh.2022.881303 (PMC9325992; doi:10.3389/fpubh.2022.881303)

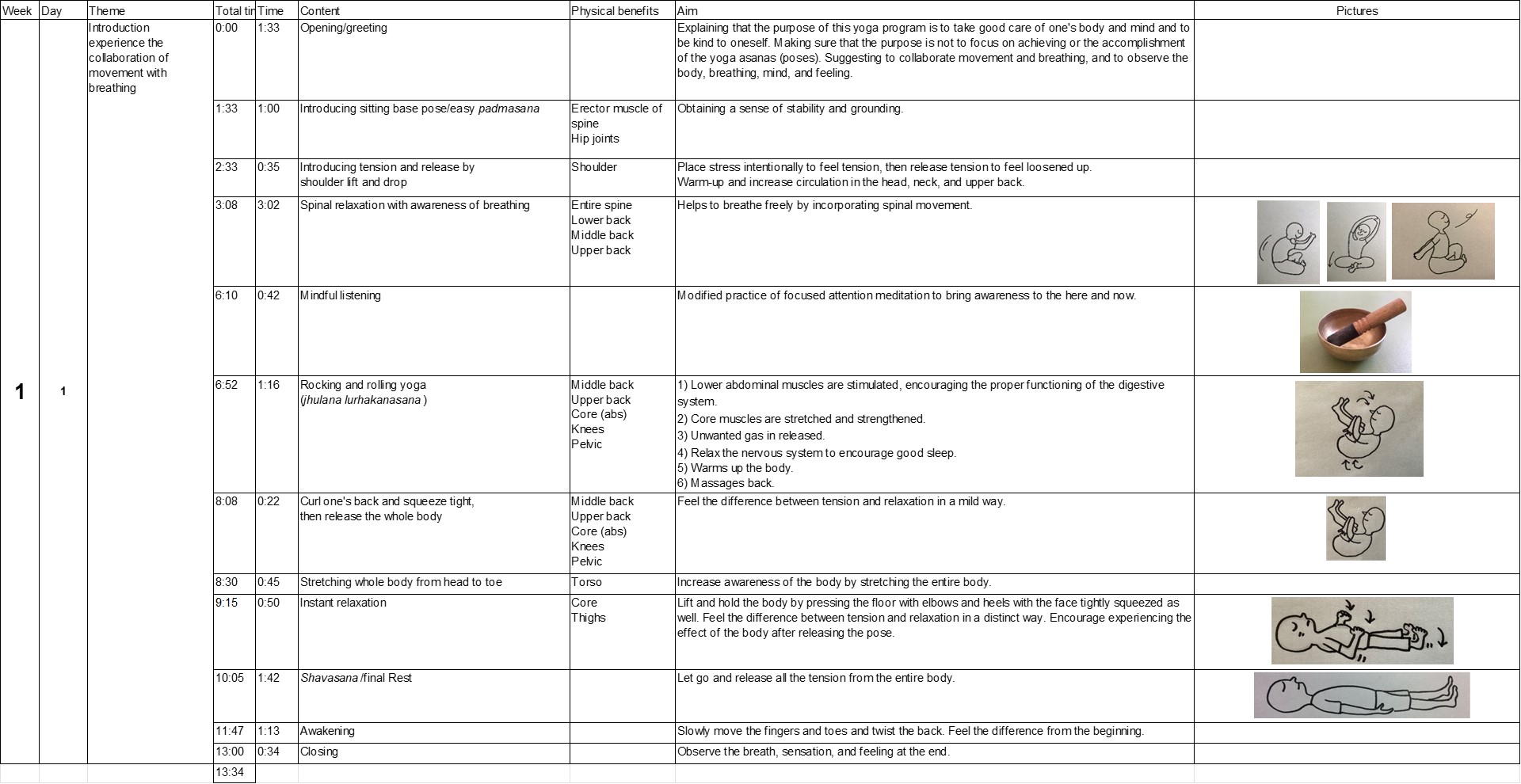

Supplement: Supplementary Figure 1A — Contents of the first day of the first week of the program. [file Data_Sheet_1.zip › Supplementary figure 1a.JPEG]

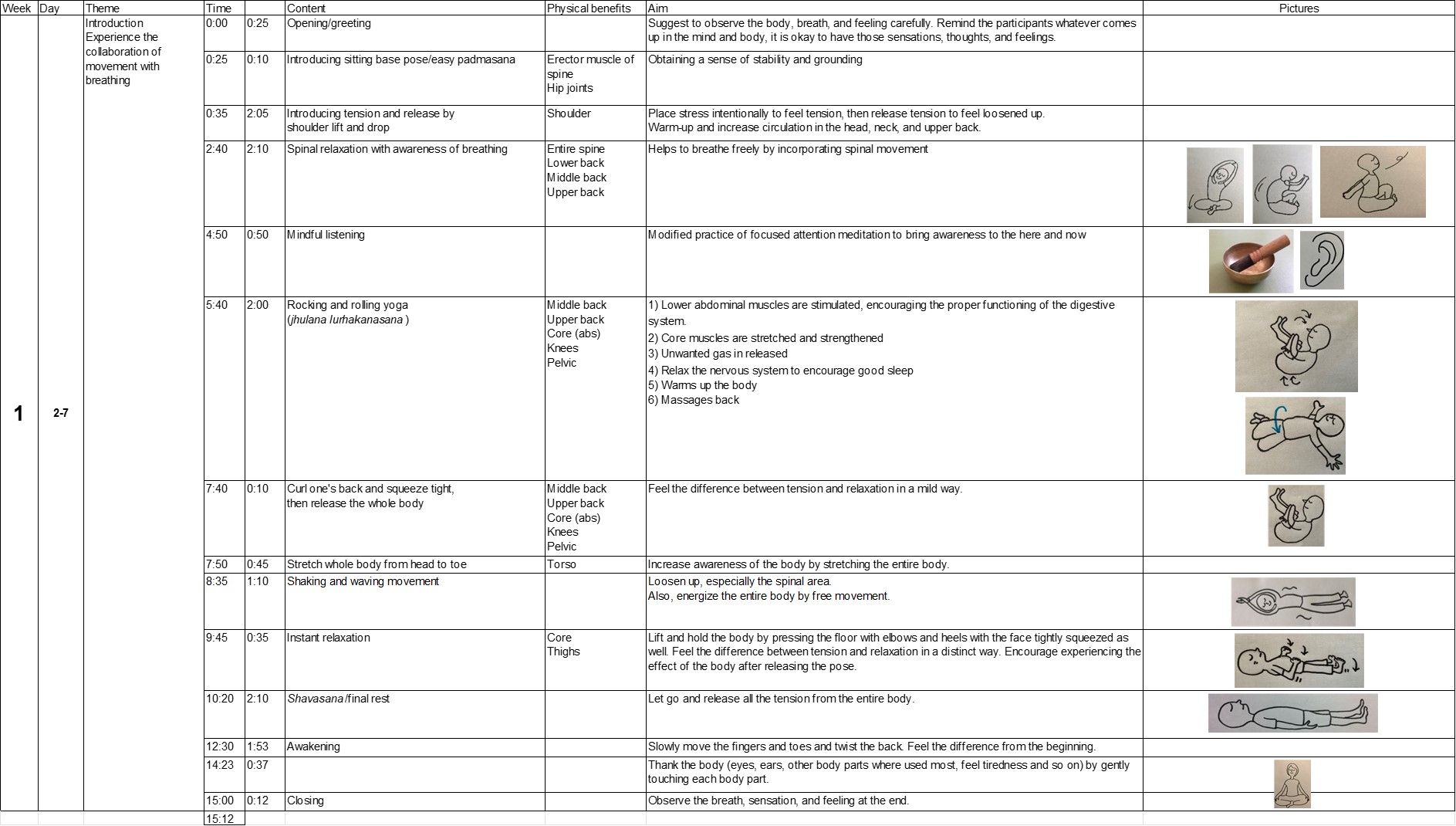

Supplement: Supplementary Figure 1A — Contents of the first day of the first week of the program. [file Data_Sheet_1.zip › Supplementary figure 1b.JPEG]

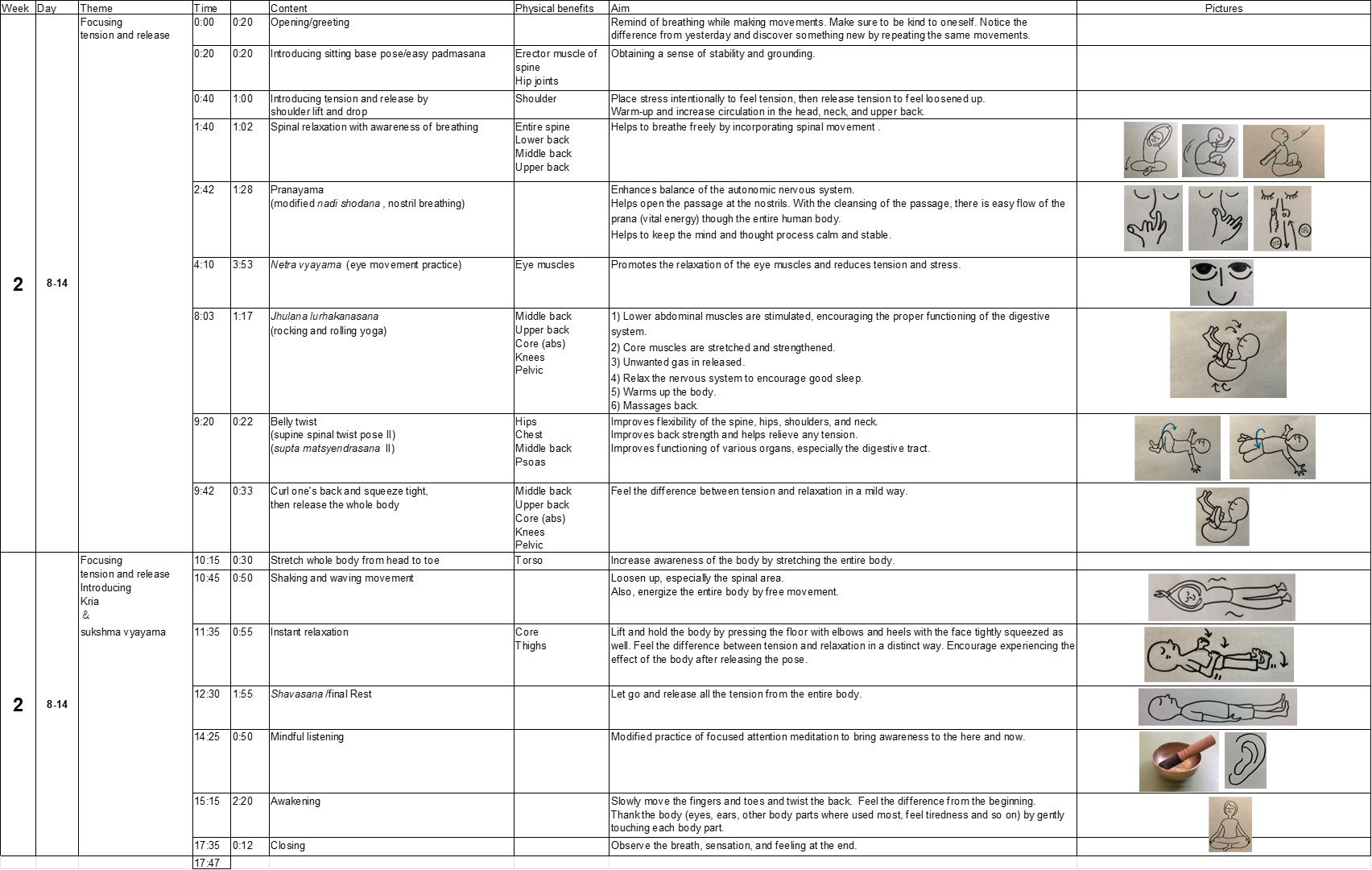

Supplement: Supplementary Figure 1A — Contents of the first day of the first week of the program. [file Data_Sheet_1.zip › Supplementary figure 1c.JPEG]

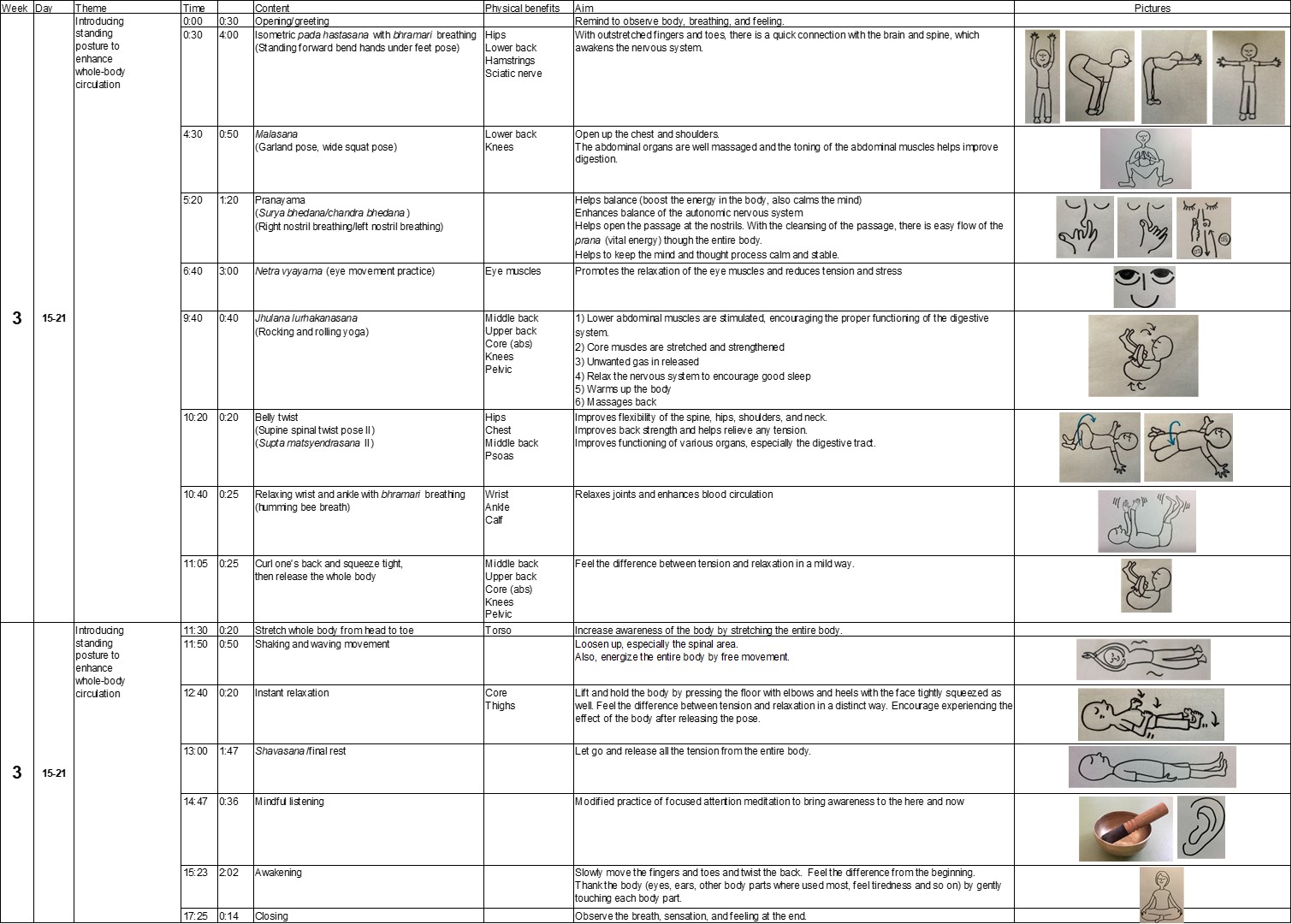

Supplement: Supplementary Figure 1A — Contents of the first day of the first week of the program. [file Data_Sheet_1.zip › Supplementary figure 1d.JPEG]

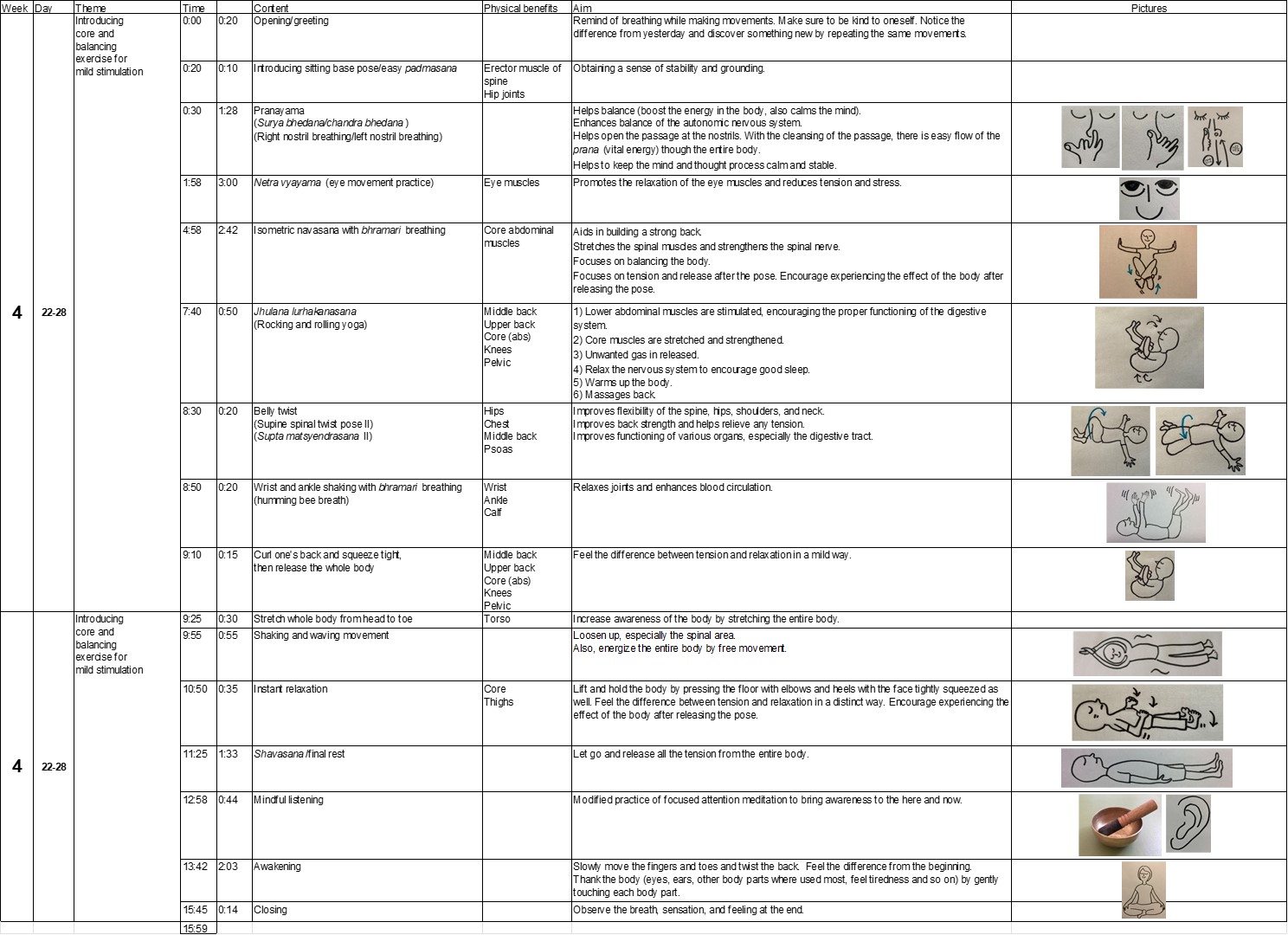

Supplement: Supplementary Figure 1A — Contents of the first day of the first week of the program. [file Data_Sheet_1.zip › Supplementary figure 1e.JPEG]

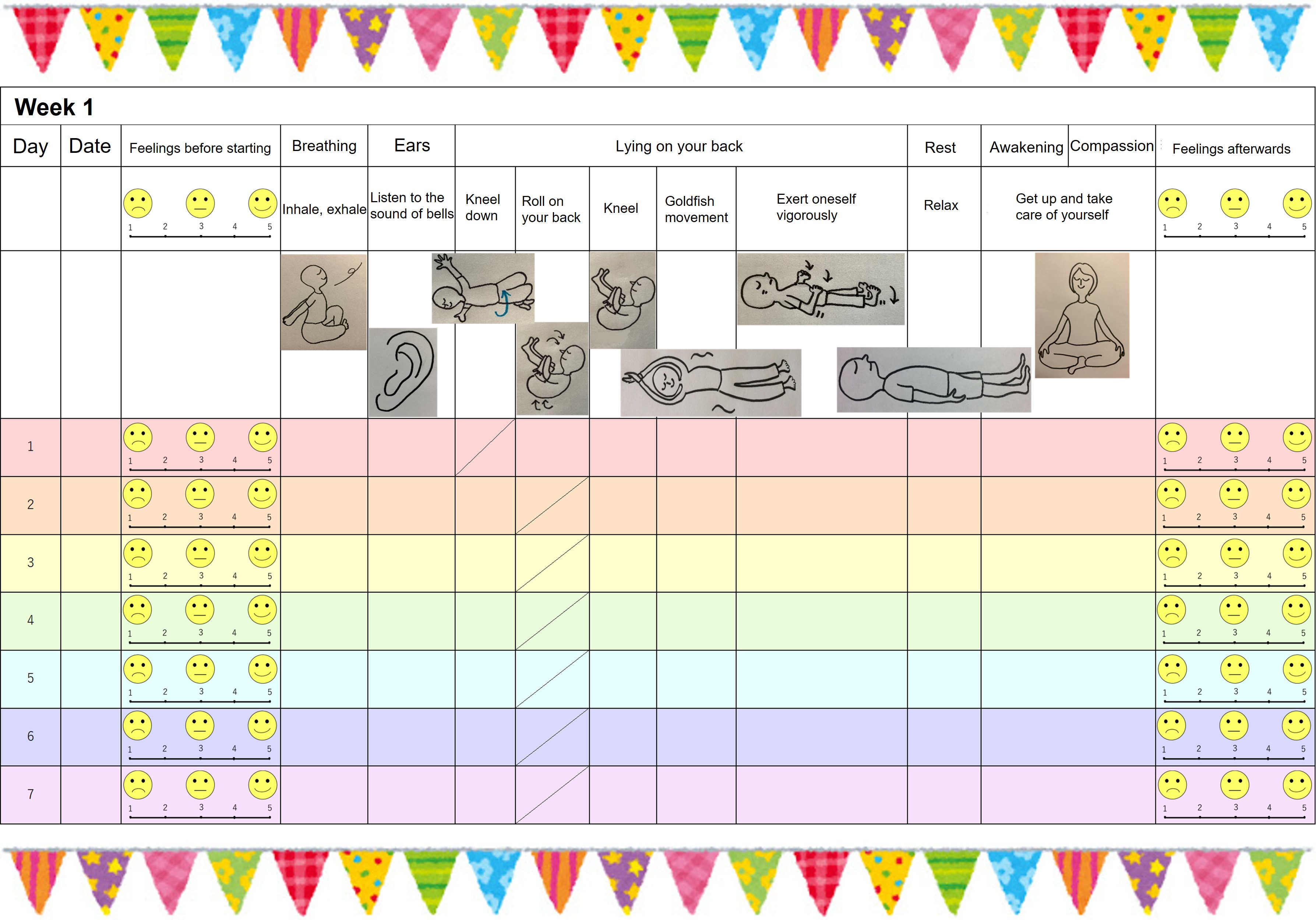

Supplement: Supplementary Figure 1A — Contents of the first day of the first week of the program. [file Data_Sheet_1.zip › Supplementary figure 2a.JPEG]

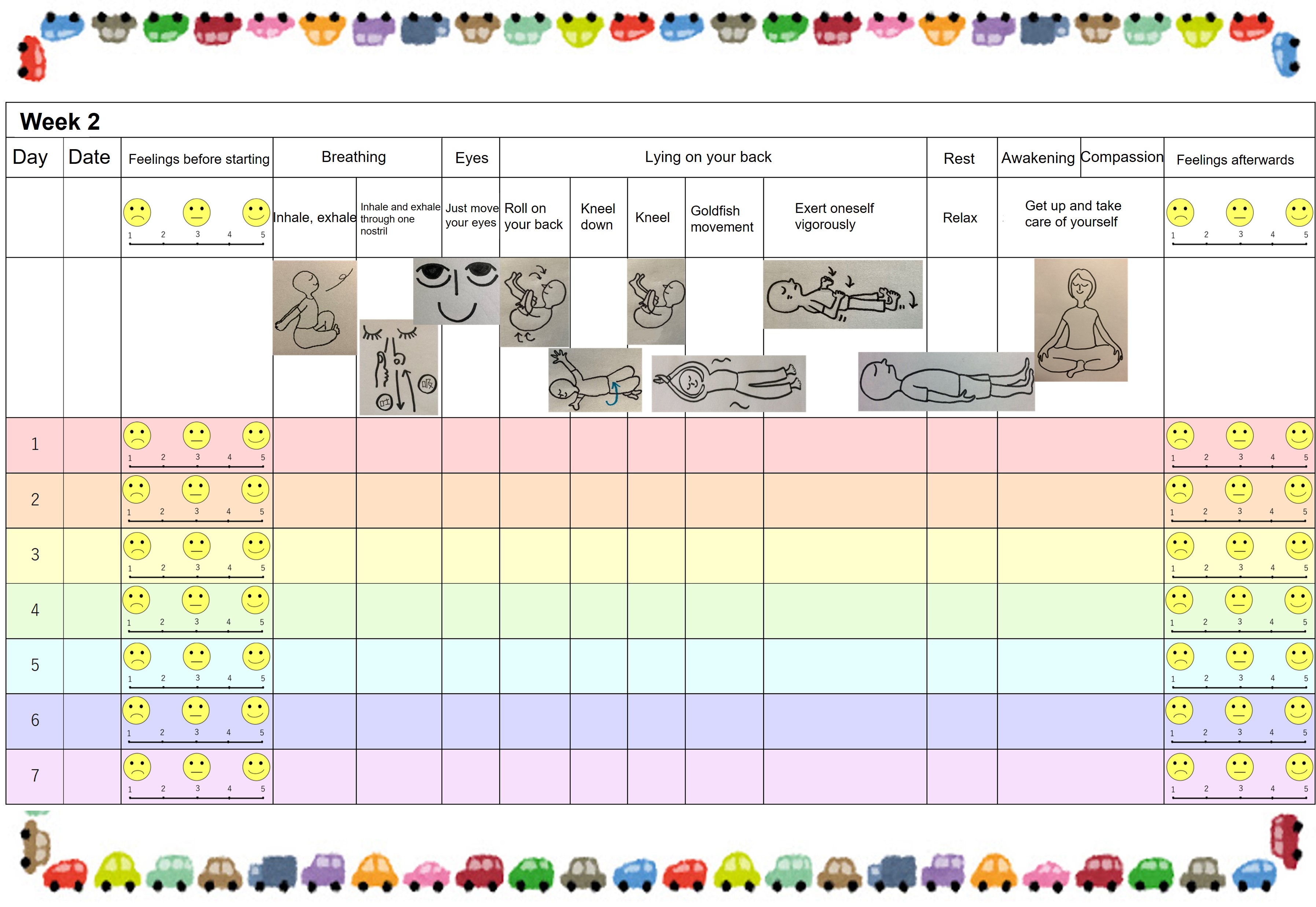

Supplement: Supplementary Figure 1A — Contents of the first day of the first week of the program. [file Data_Sheet_1.zip › Supplementary figure 2b.JPEG]

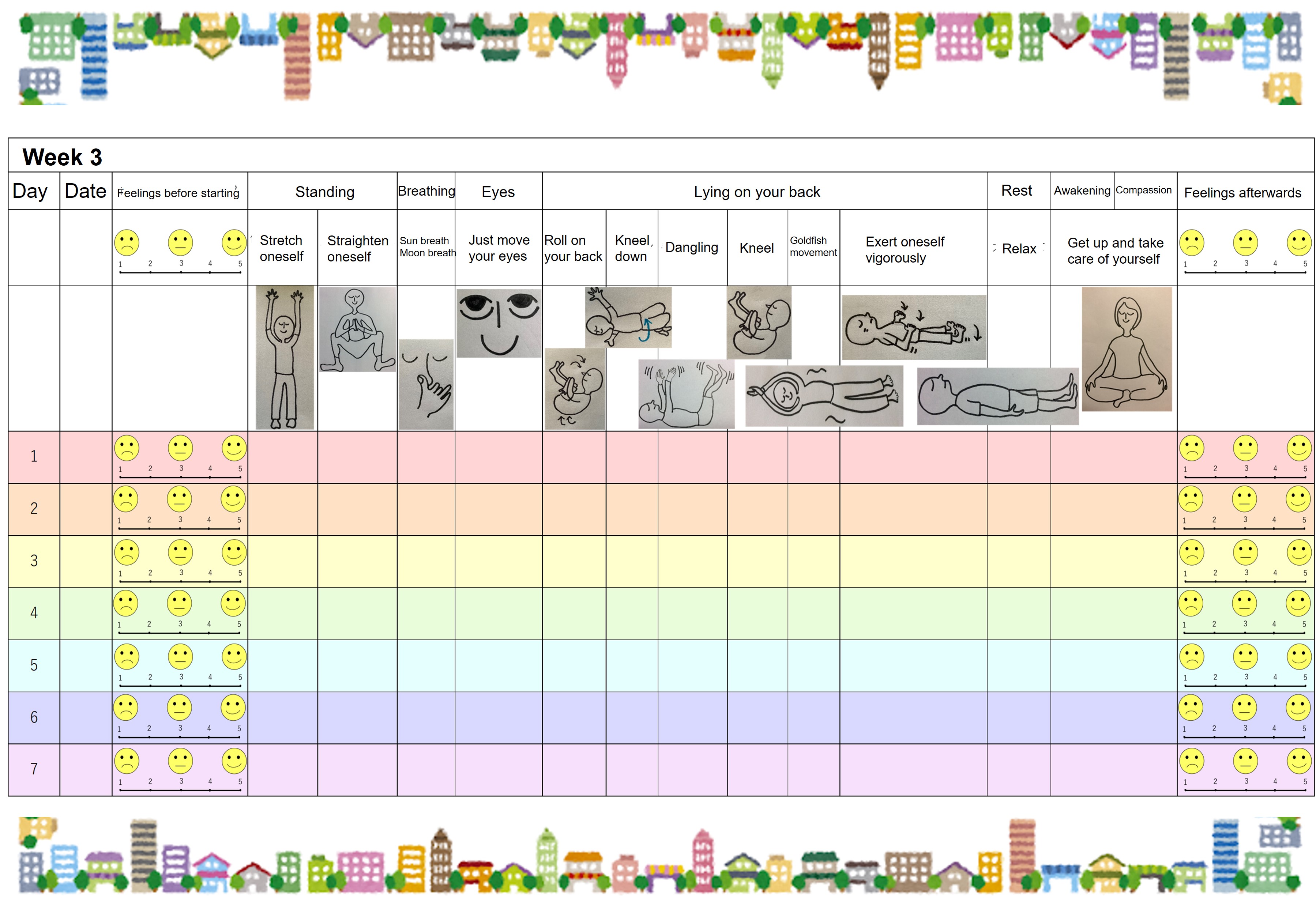

Supplement: Supplementary Figure 1A — Contents of the first day of the first week of the program. [file Data_Sheet_1.zip › Supplementary figure 2c.JPEG]

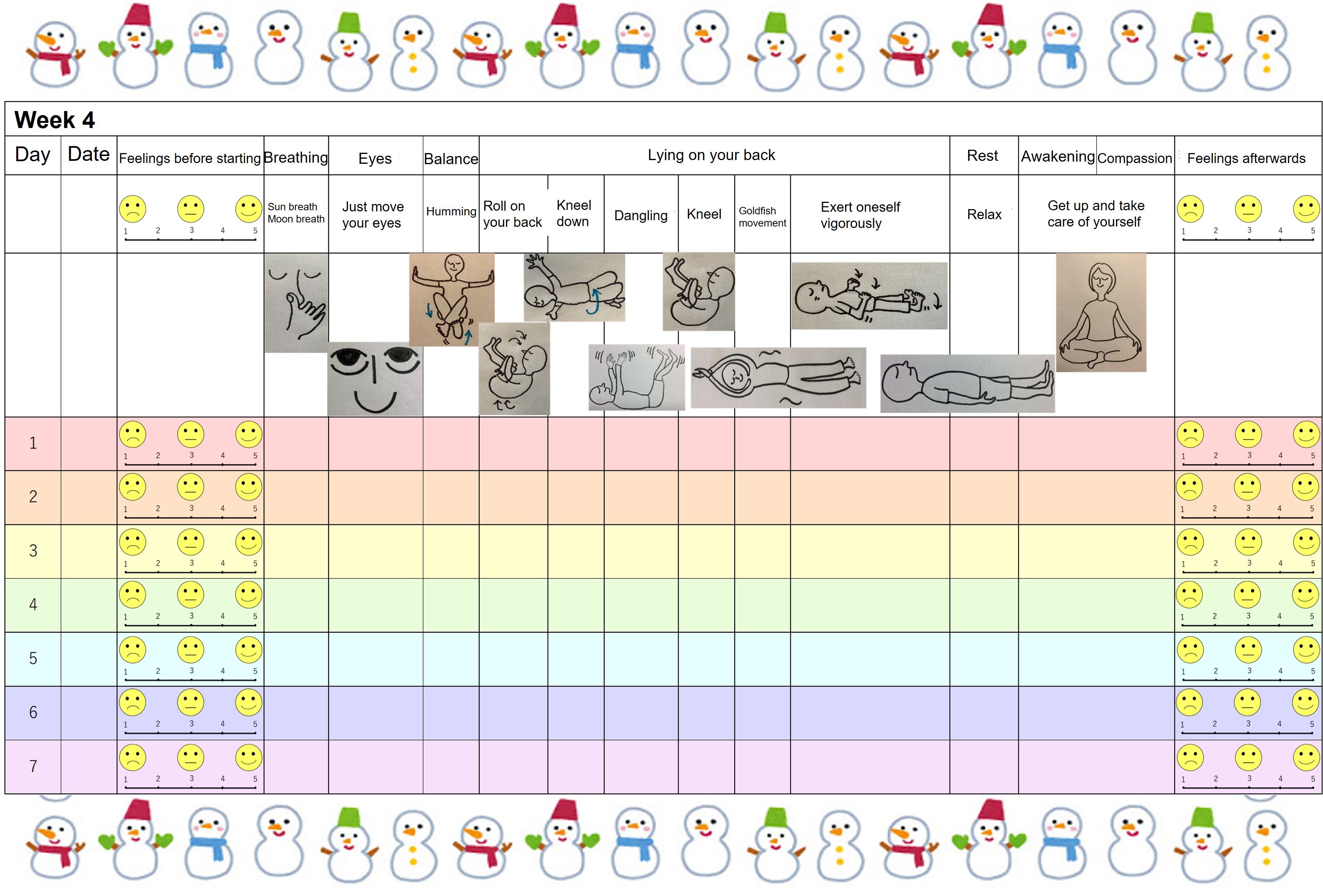

Supplement: Supplementary Figure 1A — Contents of the first day of the first week of the program. [file Data_Sheet_1.zip › Supplementary figure 2d.JPEG]

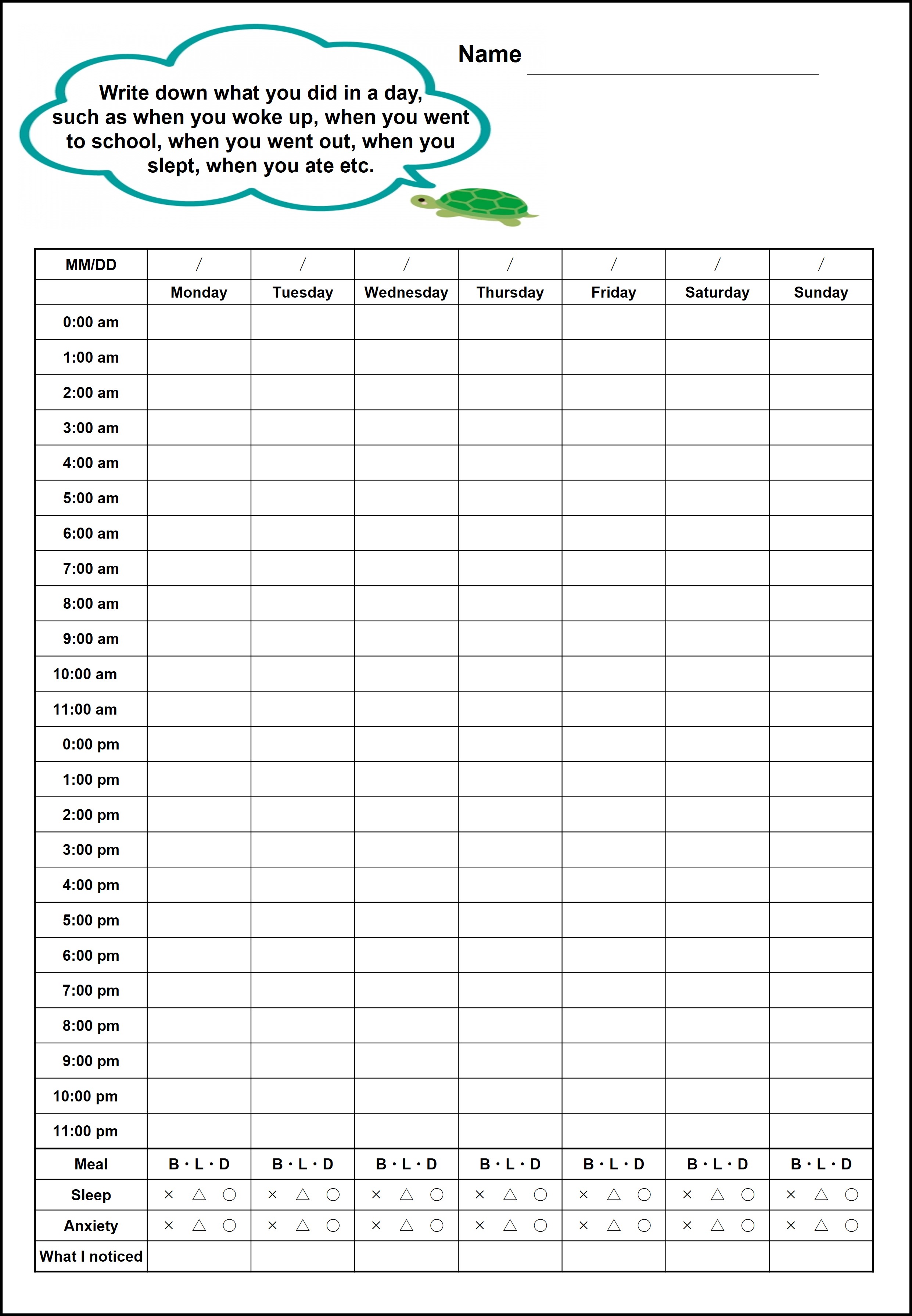

Supplement: Supplementary Figure 1A — Contents of the first day of the first week of the program. [file Data_Sheet_1.zip › Supplementary figure 3.JPEG]
